# Dysregulated circular RNAs as novel biomarkers in esophageal squamous cell carcinoma: a meta‐analysis

**DOI:** 10.1002/cam4.3703

**Published:** 2021-10-26

**Authors:** Chengcheng Guo, Jianqiang Mi, Haike Li, Panke Su, He Nie

**Affiliations:** ^1^ Department of Pathology The First Affiliated Hospital, and College of Clinical Medicine of Henan University of Science and Technology Luoyang China; ^2^ Faculty of Basic Medicine Henan Vocational College of Tuina Luoyang China; ^3^ Department of Nuclear Medicine The First Affiliated Hospital, and College of Clinical Medicine of Henan University of Science and Technology Luoyang China

**Keywords:** circular RNA, diagnoses, esophageal squamous cell carcinoma, meta‐analysis, prognoses

## Abstract

**Introduction:**

Circular RNAs (circRNAs) play critical roles in tumorigenesis, but their clinical efficacy in esophageal squamous cell carcinoma (ESCC) still retains controversial. This meta‐analysis aims at evaluating the associations between circRNA expressions and clinicopathologic features as well as the diagnostic and prognostic values of circRNAs in ESCC.

**Materials & Methods:**

PubMed, EMBASE, and other online databases were systematically searched to collect studies on circRNAs and clinicopathological features, diagnostic, and/or prognostic assessments of ESCC. The quality of included studies was evaluated using the Quality Assessment of Diagnostic Accuracy Studies 2 (QUADAS‐2) and Newcastle‐Ottawa Scale (NOS) scales. The included studies were quantitatively weighted and merged, and diagnostic indicators, hazard ratios (HRs) and the corresponding 95% confidence intervals (CIs) were calculated. *P* values were merged by Fisher᾽s method. Sources of heterogeneity were traced using subgroup, sensitivity, and meta‐regression analyses.

**Results:**

As a result, 12 studies were included, representing 769 ESCC patients. The meta‐analysis showed that abnormal expressions of circRNAs were associated to TNM stage as well as lymph node and distant metastases in ESCC cases. CircRNA was used to distinguish ESCC patients from healthy controls, and the merged sensitivity, specificity, and the area under the curve (AUC) of ESCC were 0.78 (95% CI: 0.74–0.81), 0.79 (95% CI: 0.75–0.83), and 0.86, respectively. The survival analysis showed that upregulated oncogenic circRNA levels in ESCC tissues was associated with the shorter overall survival (OS) of the patients (univariate analysis: HR = 2.25, 95% CI: 1.71–2.95, *p* = 0.000, *I* ^2 ^= 0.0%; multivariate analysis: HR = 2.50, 95% CI: 1.61–3.89, *p* = 0.000, *I* ^2^ = 0.0%), while the OS of ESCC patients presenting overexpressions of tumor‐suppressive circRNAs was significantly ameliorated (HR = 0.29, 95% CI: 0.20–0.42, *p* = 0.000, *I* ^2^ = 0.0%). The subgroup analyses based on circRNA biofunctions, sample size, and reference gene also revealed robust results.

**Conclusion:**

CircRNAs can be used as promising molecular biomarkers for the early diagnosis and prognosis monitoring of ESCC.

## Graphical abstract

CircRNAs have been highlighted as novel biomarkers for the management of esophageal squamous cell carcinoma (ESCC), but the findings remain controversial. The present meta‐analysis sought to evaluate the clinicopathologic, diagnostic and prognostic significance of circRNA expression in ESCC.

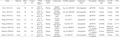

## 1 BACKGROUND

Esophagus carcinoma (EC) is one of the most common malignant tumors in the world, and mainly can be categorized into two types: ESCC and esophageal adenocarcinoma (EA).^1^ China is home to EC cases with a high incidence. Specifically, about 70% of total EC patients across the world are in China, and some 90% of whom are diagnosed as ESCC according to the pathological type.^2^, ^3^ As with the latest statistics of cancer reports in China, the morbidity rate of ESCC ranks the fourth, while its death rate ranks the sixth among all cancers.^4^ Because of the less obvious symptoms at the early stage, the early diagnosis rate is low. Moreover, 50% of ESCC patients cannot get access to timely surgical resection, and the 5‐year survival rate is less than 20%.^2^ Currently, cytokeratin 19 fragment (CYFRA 21–1), squamous cell carcinoma antigen (SCC‐Ag), carcinoembryonic antigen (CEA), and carbohydrate antigen 19–9 (CA19‐9) have been utilized as common serum tumor markers of ESCC, but these routine biomarkers have multiple shortcomings such as low detection sensitivity and susceptibility to environmental factors.^5^, ^6^ Therefore, the priority is to confirm effective molecular markers for a higher diagnosis rate of early ESCC with the improved prognosis.

Circular RNA (circRNA) as a type of coding/non coding RNA that can covalently bind its 3᾽ and 5᾽ ends to form a closed loop is widely expressed in mammalian cells, featuring tissue‐cell specificity, structural stability, and sequence conservation.^7^, ^8^ It has been confirmed that circRNA is mainly formed by exons and exists in a large number of eukaryotic cells.^9^ CircRNA contains more transcripts than linear mRNA, which means circRNA can regulate more bioactivities at the transcriptional and posttranscriptional levels.^10^, ^11^ CircRNA, as a component of competitive ceRNA, also plays a critical role in cell cycle or senescence by inhibiting the activity of miRNA and regulating gene transcription, translation, and other functions.^12^, ^13^ The involvement of circRNA in the occurrence and development of malignant tumors as shown in recent studies underpins its diagnostic and prognostic values especially in ESCC.^14^, ^15^, ^16^, ^17^, ^18^, ^19^, ^20^, ^21^, ^22^, ^23^, ^24^, ^25^, ^26^, ^27^ As circRNA is not sensitive to nuclease and is more stable than ordinary linear RNA, it is expected to become a new biomarker of ESCC.^13^, ^15^, ^21^, ^23^, ^24^, ^25^, ^27^ Small sample size, single population, large result bias, single institutional studies, and many others are existing defects that thwart the verification of such efficacy of circRNAs. This study aimed at systematically evaluating potential application values of circRNA profiling in the diagnosis and prognosis monitoring of ESCC using the quantitative meta‐analysis.

## 2 METHODS

### 2.1 Literature search

Two authors independently searched PubMed, EMBASE, BioMed Central, Web of Science, CNKI and other online databases, and collected English‐language literature published through January 31, 2020. The search terms encompassed esophageal squamous cell carcinoma, esophagus cancer, circRNA, circular RNA, hsa circ, clinicopathologic feature, clinicopathological characteristics, clinical factor, diagnoses, diagnosis, sensitivity, specificity, ROC curve, area under the curve, AUC, prognosis, prognoses, survival, overall survival (OS), progression free survival (PFS), disease free survival (DFS), relapse free survival (RFS), and HR.

### 2.2 Inclusion and exclusion criteria

The inclusion criteria were defined as follows: (a) case‐control studies on the correlation between circRNA expressions and clinicopathological characteristics, diagnosis and/or prognosis of ESCC; (b) studies with TP, FP, FN, TN, and other indices that could be directly obtained or calculated indirectly from diagnostic studies; and (c) with indicators of prognostic studies, comprising OS, PFS, DFS, and/or RFS, HR values and 95% CIs. The exclusion criteria were as follows: (a) the data extraction that was not enough to build a 2 × 2 four‐fold table, or HR and 95% CI could not be obtained, both directly and indirectly; (b) a small number of included subjects of was less than 20 or studies that were evaluated as low‐quality; and (c) the following types of studies including basic studies, reviews, meeting abstract, etc.

### 2.3 Data extraction

Data extraction was completed by two authors independently, and the extracted information including: first author, publication date, research population, the number of cases, clinical stages, detection methods, circRNA type, expression levels, *p* values of the correlation analyses between circRNAs and clinicopathological characteristics, reference gene, cut‐off setting, sensitivity, specificity, survival time, HR and the corresponding 95%CI, follow‐up period, etc.

### 2.4 Evaluation of the methodological quality of studies

For the diagnostic studies to be included, their quality was evaluated using the QUADAS‐2 tool that consisted of seven items covering case selection, index test, golden standard, and flow and timing.^28^ The total score of ≥4 points (with a full score of 7 points) indicated that the quality of a study was high. The case‐control study was evaluated according to the NOS scale containing eight items that could be classified into case selection, comparability, exposure evaluation, or outcome evaluation.^29^ The total score of ≥5 points (with a full score of 9 points) suggested that the quality of a study was high.

### 2.5 Statistical analysis

This study was carefully carried out according to PRISMA2009 guidelines.^30^ All statistical analyses were performed using Stata 12.0 software and MetaDiSc 1.4 software. Spearman correlation coefficient was used to detect the source of heterogeneity caused by non‐threshold effect, while Cochran᾽s *Q* test and *I* ^2^ test were used to evaluate the heterogeneity caused by threshold effect. A *p* < 0.01 or *I* ^2^ > 50% indicated that there was a large heterogeneity among the studies, so a random‐effect model was adopted to merge the data, otherwise a fixed‐effect model would be used. The merged effect‐size indicators comprised sensitivity, specificity, positive likelihood ratio (PLR), negative likelihood ratio (NLR), diagnostic odds ratio (DOR), AUC, HR, and the corresponding 95% CI. The *p* values of the correlations between circRNA levels and clinicopathological characteristics of ESCC were merged using Fisher᾽s method.^31^ Then, subgroup, sensitivity and meta‐regression analyses were conducted to explore the causes of between‐study heterogeneity. The publication bias between studies was evaluated by Deek᾽s funnel plot, visual Funnel plot, Begg's and Egger's tests. A *p* < 0.05 was considered statistically significant.

## 3 RESULTS

### 3.1 Literature search results

**FIGURE 1:**
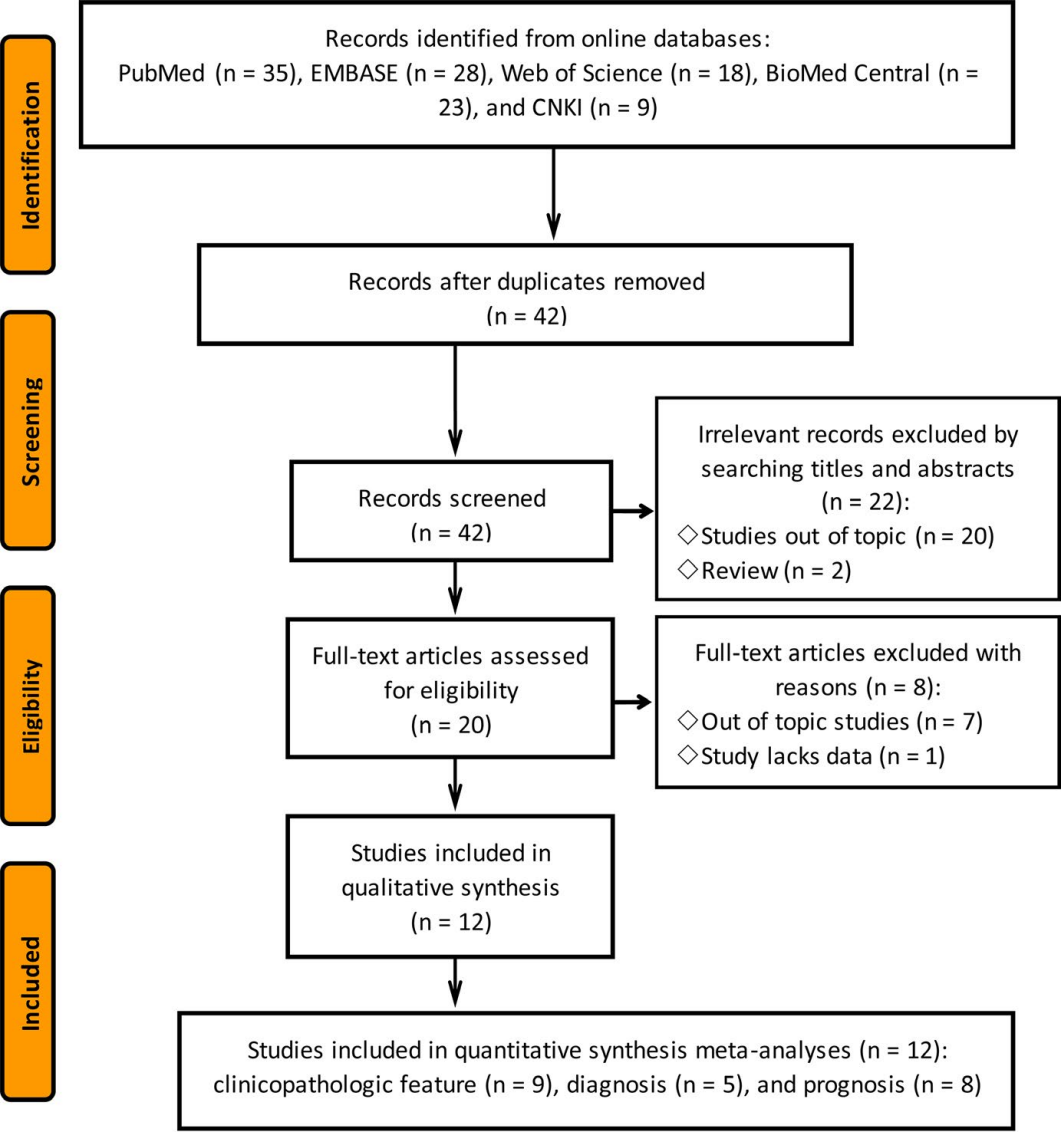
The flow chart of literature searching according to the PRISMA 2009 guidelines

After the initial retrieval, 42 studies were obtained from the databases, and 22 (including 2 reviews and 20 unrelated researches) studies were ruled out after carefully reading titles and abstracts. The remaining 20 were rigorously evaluated by reading the full texts, of which eight were identified as ineligible for they did not meet the inclusion criteria and were further excluded and 12 (9 studies with clinicopathologic feature, 5 diagnostic studies, and 8 prognosticones)^14^, ^27^ were finally included for the subsequent meta‐analyses (Figure 1).

### 3.2 Data characteristics and the methodological quality of studies

A total of 769 ESCC patients, featuring a predominant Asian population, merged from the 12 included studies were enrolled. All ESCC cases were pathologically confirmed, of whom early ESCC (stage 0, I, and II) patients in the diagnostic studies accounted for 53.14% (152/286). In the control group, only healthy controls were included in the diagnostic studies. All tissue and plasma samples were preoperatively collected before any treatment. CircRNA expression levels were detected using RT‐qPCR, with *GAPDH*, or *β*‐*Actin* as internal reference genes. Of the eight included prognostic studies, four provided HR values and 95% CIs, and four using related formulas to calculate the prognosis curve indirectly. There were 15 circRNAs involved, of which 10 (circ‐DLG1, circ‐TTC17, Circ‐SLC7A5, hsa circ 0000437, hsa circ 0004771, CiRS‐7, circrna_100876, hsa_circ_0006948, hsa_circ_0006168, and has_circ_0067934) were upregulated in ESCC, acting as oncogenes, and five (hsa_circ_0001946, hsa_circ_0062459, circ‐SMAD7, hsa_circ_0001946, and CircVRK1) tumor‐suppressive genes were downregulated. The main clinical characteristics of all included studies are shown in Tables 1 and 2.

**TABLE 1:** Main clinical characteristics in diagnostic studies

Study	Ethnicity	ESCC size	Control size	TNM stage(I, II, III, IV)	Sample source	Control type	CircRNA signature	Expression status	Test method	Reference gene	Cut‐off setting	AUC
Fan L 2019 ^15^	Asian	50	50	0–II: 21 III–IV: 29	Plasma	Healthy individuals	hsa_circ_0001946	Downregulated	qRT‐PCR	Unclear	Unclear	0.894
Fan L 2019 ^15^	Asian	50	50	0–II: 21 III–IV: 29	Plasma	Healthy individuals	hsa_circ_0062459	Downregulated	qRT‐PCR	Unclear	Unclear	0.836
Rong J 2018 ^21^	Asian	35	28	I–II: 24; III–IV: 11	Plasma	Normal cases	circ‐DLG1	Upregulated	qRT‐PCR	GAPDH	−4.924	0.648
Wang Q 2019 ^24^	Asian	30	25	I–II: 15; III–IV: 15	Plasma	Normal Control	circ‐TTC17	Upregulated	qRT‐PCR	GAPDH	−2.548	0.820
Wang Q 2020 ^23^	Asian	87	53	I–II: 36; III–IV: 51	Plasma	Healthy individuals	Circ‐SLC7A5	Upregulated	qRT‐PCR	GAPDH	Unclear	0.7717
Huang E 2020 ^18^	Asian	105	105	I–II: 40; III–IV: 65	Plasma	Healthy control	hsa circ 0000437	Upregulated	qRT‐PCR/ 2^–△△^Ct method	β‐Actin	Median of expression	0.672
Huang E 2020^18^	Asian	105	105	I–II: 40; III–IV: 65	Plasma	Healthy control	hsa circ 0004771	Upregulated	qRT‐PCR/ 2^–△△^Ct method	β‐Actin	Median of expression	0.816
Zhang Y 2019 ^25^	Asian	32	25	I–II: 16; III–IV: 16	Plasma	Healthy individuals	circ‐SMAD7	Downregulated	qRT‐PCR/ΔCt method	GAPDH	Unclear	0.859

Abbreviations: AUC, area under the curve; CircRNA, circular RNA; GAPDH, reduced glyceraldehyde‐phosphate dehydrogenase; ESCC, esophageal squamous cell carcinoma; qRT‐PCR, quantitative reverse transcription‐polymerase chain reaction.

**TABLE 2:** Characteristics of the included studies for prognosis and clinicopathologic features

Included study	Ethnicity	Sample Type	CircRNA signature	ESCC case			Expression Status	Survival Indicator	Follow‐up time	HR & 95% CI Extraction	*P* Value (Survival)
				Total	CircRNA high	CircRNA low					
Li RC 2018 ^19^	Asian	Tissue	CiRS−7	123	61	62	Increased	OS	Mentioned but unclear	Directly	0.000
Li RC 2018 ^19^	Asian	Tissue	CiRS−7	123	61	62	Increased	DFS	Mentioned but unclear	Directly	0.000
Cao S 2020 ^14^	Asian	Tissue	circrna_100876	74	37	37	Increased	OS	Not mentioned	Indirectly	0.021
Cao S 2020 ^14^	Asian	Tissue	circrna_100876	74	37	37	Increased	RFS	Not mentioned	Indirectly	0.029
Fan L 2019 ^15^	Asian	Tissue	hsa_circ_0001946	50	Unclear	Unclear	Decreased	OS	Not mentioned	Directly	All with *p* < 0.05
Fan L 2019 ^15^	Asian	Tissue	hsa_circ_0001946	50	Unclear	Unclear	Decreased	DFS	Not mentioned	Directly	All with *p* < 0.05
He Y 2019 ^17^	Asian	Tissue	CircVRK1	88	46	42	Decreased	OS	Not mentioned	Indirectly	0.035
Wang Q 2020 ^23^	Asian	Tissue	Circ‐SLC7A5	87	69	18	Increased	OS	Not mentioned	Indirectly	0.0079
Wang Q 2019 ^24^	Asian	Tissue	Circ‑TTC17	30	22	8	Increased	OS	Two years (until December 2017)	Indirectly	0.01
Pan Z 2019 ^20^	Asian	Tissue	Hsa_circ_0006948	153	128	25	Increased	OS	Not mentioned	Directly	<0.0009
Huang E 2020 ^18^	Asian	Tissue	hsa circ 0004771	105	53	52	Increased	OS	Not mentioned	Directly	0.009
Huang E 2020 ^18^	Asian	Tissue	hsa circ 0004771	105	53	52	Increased	DFS	Not mentioned	Directly	0.006
Shi Y 2019 ^22^	Asian	Tissue	hsa_circ_0006168	52	26	26	Increased	/	/	/	/
Xia W 2016 ^27^	Asian	Tissue	has_circ_0067934	51	Unclear	Unclear	Increased	/	/	/	/

CircRNA, circular RNA; DFS, disease free survival; HR, hazard ratio; OS, overall survival; RFS, relapse free survival.

The quality of the included studies was strictly evaluated using the QUADAS‐2 and NOS scales. It was found that the cumulative scores of the diagnostic studies were ≥5 points, and those of the observation studies were ≥6 points, suggesting that the overall methodological quality of the studies was high (Tables 3 and 4).

**TABLE 3:** Quality bias of the diagnostic studies using the QUADAS‐2 checklist

Study	Risk of bias				Concerns regarding applicability			Summed quality scores
	Patient selection	Index test	Reference standard	Flow and timing	Patient selection	Index test	Reference standard	
Fan L 2019 ^15^	Low	Low	Low	Unclear	Low	Unclear	Low	5
Rong J 2018 ^21^	Low	Low	Low	Unclear	Low	Low	Low	6
Wang Q 2019 ^24^	Low	Low	Low	Unclear	Low	Low	Low	6
Wang Q 2020 ^23^	Low	Low	Low	Unclear	Low	Unclear	Low	5
Huang E 2020 ^18^	Low	Low	Low	Unclear	Low	Low	Low	6
Zhang Y 2019 ^25^	Low	Low	Low	Unclear	Low	Low	Low	6

QUADAS, Quality Assessment for Studies of Diagnostic Accuracy.

**TABLE 4:** Quality bias of the prognostic studies using the NOS checklist

Included study	Cohort selection				Comparability Comparability of cases and controls on the basis of the design or analysis	Outcome ascertainment			Summed quality scores
	Representativeness of the exposed cohort	Selection of the non‐exposed cohort	Ascertainment of exposure	Demonstration that outcome of interest was not present at start of study		Assessment of outcome	Was follow‐up long enough for outcomes to occur	Adequacy of follow up of cohorts	
Li RC 2018 ^19^	1	1	1	1	1	1	1	1	8
Cao S 2020 ^14^	1	1	1	1	1	1	0	0	6
Fan L 2019 ^15^	1	1	1	1	1	1	0	0	6
He Y 2019 ^17^	1	1	1	1	1	1	0	0	6
Wang Q 2020 ^23^	1	1	1	1	1	1	0	0	6
Wang Q 2019 ^24^	1	1	1	1	1	1	1	1	8
Pan Z 2019 ^20^	1	1	1	1	1	1	0	0	6
Huang E 2020 ^18^	1	1	1	1	1	1	0	0	6

NOS, Newcastle Ottawa Scale.

### 3.3 Correlations between circRNA expressions and clinicopathological characteristics of ESCC

Abnormal circRNA expressions were correlated with TNM stage (chi^2^ = 61.64, *p* = 0.000), lymph node metastasis (chi^2^ = 35.06, *p* = 0.000), distant metastasis (chi^2^ = 16.40, *p* = 0.012), and Cyfra21‐1 level (chi^2^ = 18.23, *p* = 0.006) in ESCC patients, but not significant in age, gender, tumor size, smoking status, as well as CEA and AFP levels, all with *p* > 0.05 (Table 5).

**TABLE 5:** Associations between circRNA expression and clinicopathological parameters in patients having ESCC

Clinicopathological parameters	Included studies	Chi^2^ value	Pooled *P* value
Age (>60 vs. 60)	10	13.07	0.874
Gender	10	17.42	0.626
Tumor size	8	14.73	0.545
Differentiation status	10	16.89	0.661
TNM stage	7	61.64	0.000
Lymph node metastasis	6	35.06	0.000
Distant metastasis	3	16.40	0.012
CEA	4	13.87	0.085
Cyfra21‐1	3	18.23	0.006
AFP	2	4.24	0.375
Smoking status	2	4.66	0.324

### 3.4 Analyses for the diagnostic efficacy of circRNAs

**FIGURE 2:**
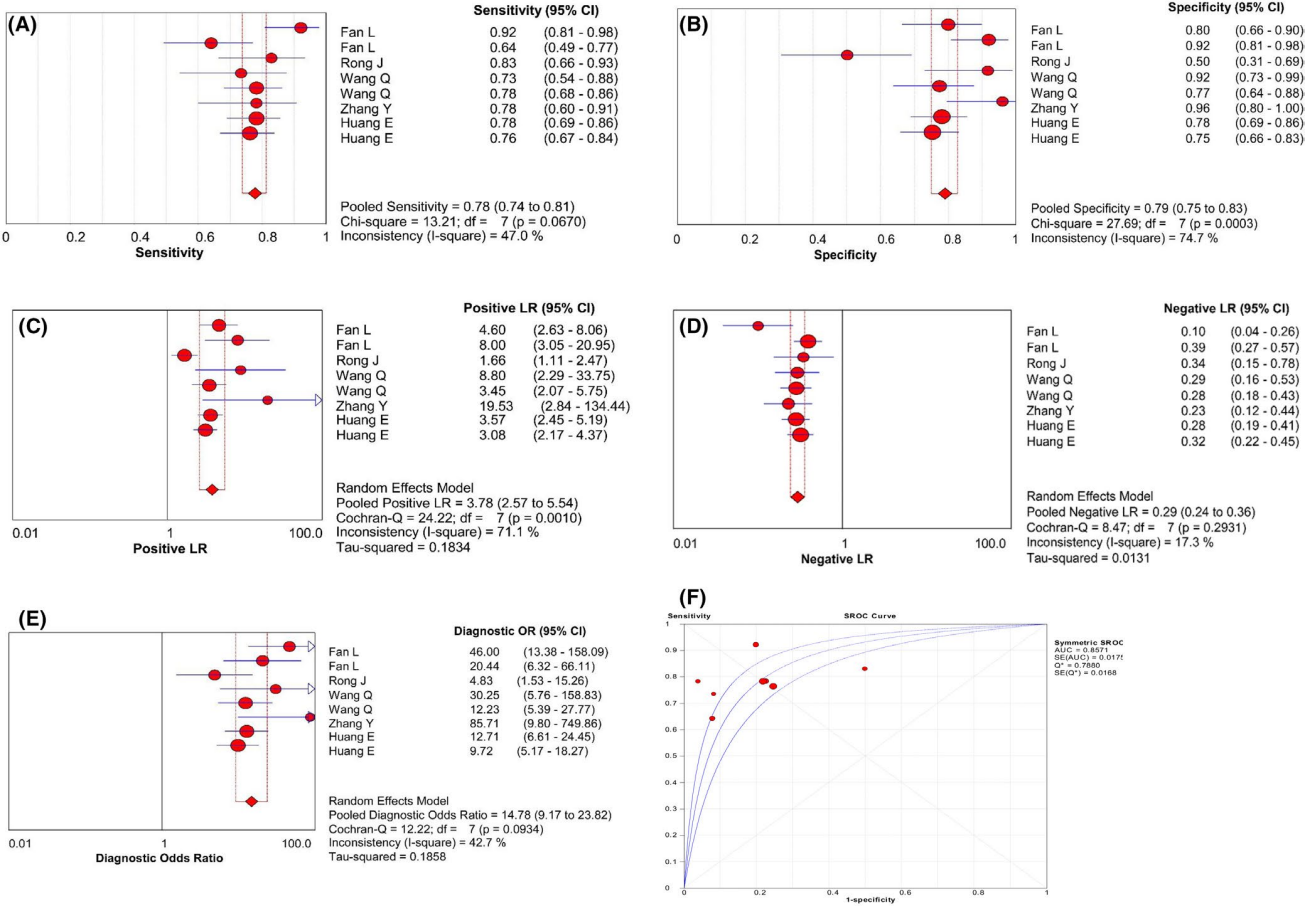
The pooled diagnostic indicators of circRNAs in diagnosing ESCC. (A) Sensitivity, (B) specificity, (C) PLR, (D) NLR, (E) DOR, and (F) AUC

CircRNA levels from six studies were evaluated for understanding the diagnostic efficacy in ESCC. The heterogeneity test showed minor heterogeneity existing in the overall diagnostic effect (*I* ^2^ = 42.7%, *p* = 0.0934; Spearman correlation coefficient: 0.381, *p* = 0.352). The combined AUC of circRNAs for diagnosing ESCC was 0.86, with the corresponding sensitivity, specificity, PLR, NLR and DOR of 0.78 (95% CI: 0.74–0.81), 0.79 (95% CI: 0.75–0.83), 3.78 (95% CI: 2.57–5.54), 0.29 (95% CI: 0.24–0.36) and 14.78 (95% CI: 9.17–23.82), respectively (Figure 2). This indicated that circRNAs had high diagnostic efficiency in distinguishing ESCC patients from healthy controls. The subgroup analysis showed that the diagnostic efficacy of the downregulated circRNAs was better than that of the upregulated circRNAs (AUC: 0.93 vs. 0.84), and the diagnostic efficacy of circRNA profiling was improved when the sample size was ≥70 (AUC: 0.89 vs. 0.85). The diagnostic performance of circRNA profiling in ESCC showed no difference in the testing using different reference genes (Table 6).

**TABLE 6:** Stratified analyses of the diagnostic performance of circRNAs in ESCC

Stratified variables	Included studies	Sensitivity 95%CI	Specificity 95%CI	PLR 95%CI	NLR 95%CI	DOR 95%CI	AUC	Heterogeneity
CircRNA expression status								
Upregulated	5	0.78 (0.73–0.82)	0.76 (0.70–0.80)	3.02 (2.06–4.42)	0.30 (0.24–0.36)	11.90 (7.56–15.72)	0.84	*I* ^2^ = 0.0%; *p* = 0.4347
Downregulated	3	0.78 (0.70–0.85)	0.88 (0.81–0.93)	6.42 (3.28–12.59)	0.23 (0.10–0.50)	34.55 (15.65–76.27)	0.93	*I* ^2^ = 0.0%; *p* = 0.4382
Sample size								
≥70	3	0.78 (0.72–0.84)	0.82 (0.76–0.88)	5.37 (2.06–13.96)	0.27 (0.18–0.41)	22.46 (8.47–59.51)	0.89	*I* ^2^ = 0.0%; *p* = 0.8279
<70	5	0.77 (0.72–0.78)	0.77 (0.71–0.82)	3.33 (2.65–4.18)	0.29 (0.24–0.37)	11.33 (7.61–16.85)	0.85	*I* ^2^ = 58.3%; *p* = 0.0477
Reference gene								
*GAPDH*	4	0.78 (0.72–0.84)	0.78 (0.70–0.85)	4.22 (1.63–10.93)	0.28 (0.21–0.37)	14.99 (5.48–40.99)	0.86	*I* ^2^ = 56.1%; *p* = 0.0775
Non‐*GAPDH*	4	0.77 (0.72–0.82)	0.80 (0.75–0.84)	3.79 (2.84–5.05)	0.29 (0.20–0.41)	15.38 (8.68–27.26)	0.87	*I* ^2^ = 43.8%; *p* = 0.1484

Abbreviations: AUC, area under the curve; circRNA, circular RNA; DOR, diagnostic odds ratio; GAPDH, reduced glyceraldehyde‐phosphate dehydrogenase; NLR, negative likelihood ratio; PLR, positive likelihood ratio.

### 3.5 Prognostic efficacy of circRNAs

**FIGURE 3:**
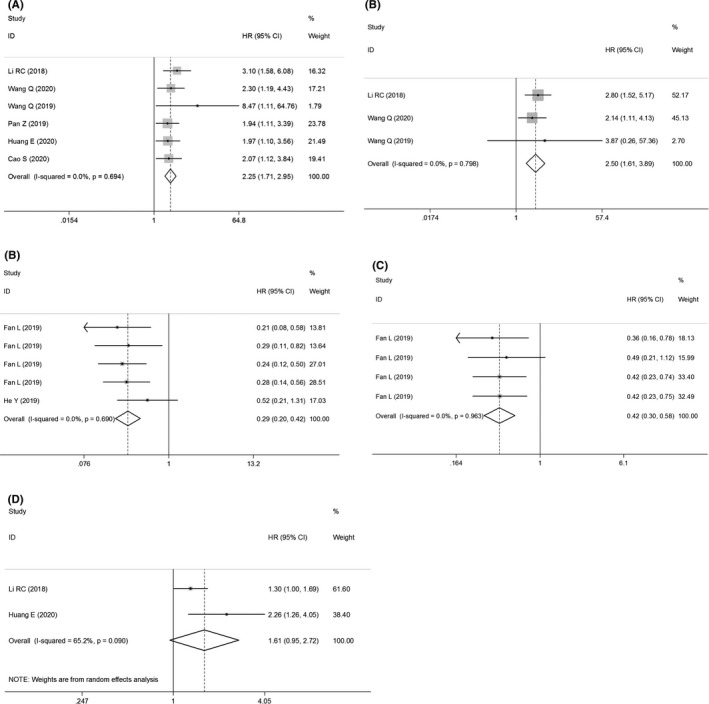
The pooled prognostic effect sizes of circRNAs in predicting the survival of ESCC. (A) the univariate analysis and (B) the multivariate analysis of oncogenic circRNAs in predicting the OS. The pooled prognostic efficacy of tumor‐suppressive circRNAs in predicting the (C) OS and (D) DFS of ESCC. (E) The combined DFS of oncogenic circRNAs

According to biofunctions of distinct types of circRNAs, they could be classified into two subgroups: oncogenic and tumor‐suppressive circRNAs. The prognosis analysis showed that overexpressions of oncogenic circRNAs were associated with shortened OS of ESCC patients (univariate analysis: HR = 2.25, 95% CI: 1.71–2.95, *p* = 0.000, *I* ^2^ = 0.0%; multivariate analysis: HR = 2.50, 95% CI: 1.61–3.89, *p* = 0.000, *I* ^2^ = 0.0%), while the OS (HR = 0.29, 95% CI: 0.20–0.42, *p* = 0.000, *I* ^2^ = 0.0%) and DFS (HR = 0.42, 95% CI: 0.30–0.58, *p* = 0.000, *I* ^2^ = 0.0%) of patients with upregulations of tumor‐suppressive circRNAs were both significantly prolonged compared with those with lowered expressions (Figure 3). However, no difference was found in the effect of oncogenic circRNAs in predicting the DFS of patients having ESCC (HR = 1.61, 95% CI: 0.95–2.72, *p* = 0.078, *I* ^2^ = 65.2%) (Figure 3).

### 3.6 Influence analysis and meta‐regression test

**FIGURE 4:**
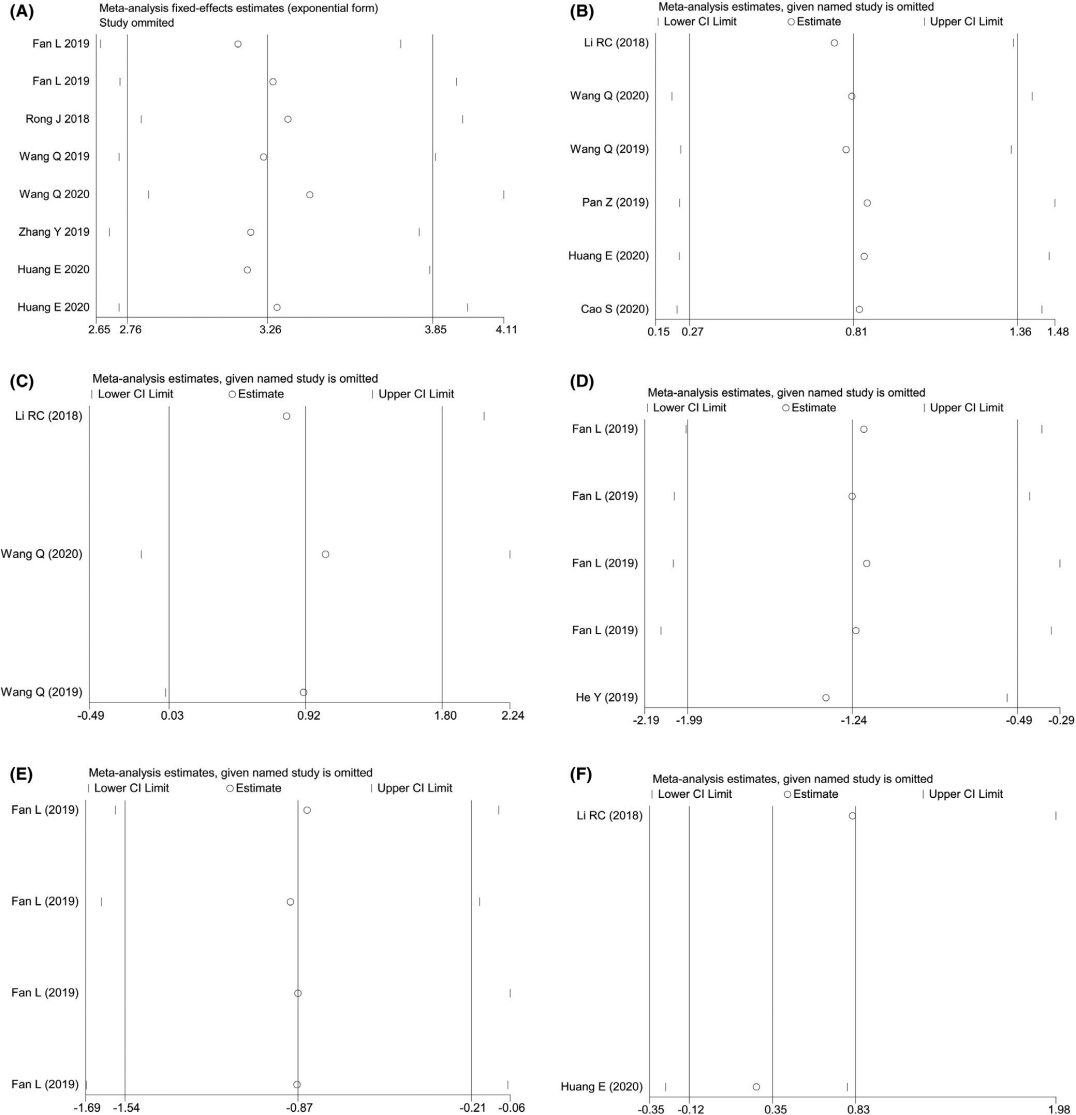
The influence analyses of the pooled effects. (A) The overall combined diagnostic effect. (B) The univariate analysis and (C) the multivariate analysis of oncogenic circRNAs in predicting the OS of ESCC patients. The pooled prognostic effect of tumor‐suppressive circRNAs in predicting the (D) OS and (E) DFS of ESCC patients. (F) The prognostic effect of oncogenic circRNAs in predicting the DFS of ESCC patients

The influence analysis showed the even distribution among studies with no deviant outliers, suggesting good homogeneity among all included studies (Figure 4). Variables for the meta‐regression test encompassed the sample size, circRNA signature, circRNA expression status, reference gene, cut‐off setting, QUADAS scores, etc. As a result, none of these variables were identified as significant factors that could cause the heterogeneity among the studies (Table 7).

**TABLE 7:** The meta‐regression analysis for the diagnostic effect

Variables	Coeff.	Std. Err.	*P* value	PDOR	95%CI
Sample size (≥100 *vs*. <100)	−0.406	0.4261	0.3846	0.67	(0.22–1.99)
CircRNA signature	−0.109	0.0730	0.1963	0.90	(0.74–1.08)
CircRNA expression status (Increased *vs*. Decreased)	−1.025	0.4754	0.0837	0.36	(0.11–1.22)
Study quality (QUADAS summed score)	−0.430	0.3917	0.3227	0.65	(0.24–1.78)
Reference gene (GAPDH *vs*. β‐Actin *vs*. others)	−0.366	0.2331	0.1770	0.69	(0.38–1.26)
Cut‐off setting (clear *vs*. unclear)	−0.572	0.3733	0.1863	0.56	(0.22–1.47)

Abbreviations: circRNA, circular RNA; Coeff, coefficient value; GAPDH, reduced glyceraldehyde‐phosphate dehydrogenase; PDOR, pooled diagnostic odds ratio; QUADAS, Quality Assessment for Studies of Diagnostic Accuracy.

### 3.7 Publication bias

**FIGURE 5:**
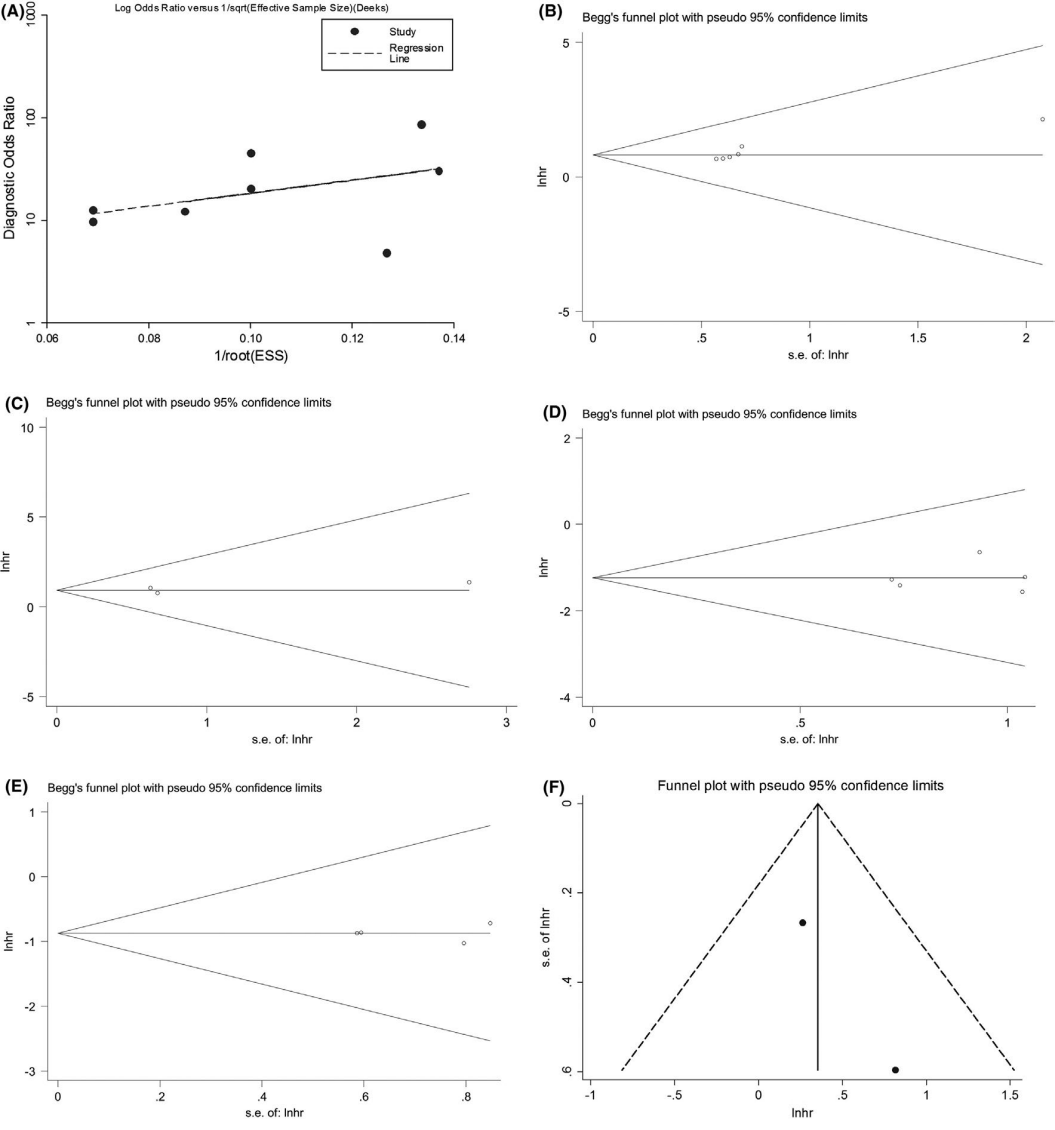
Publication bias. (A) Deek's funnel plot of the overall diagnostic effect (*p* = 0.437). Begg᾽s test for (B) the univariate analysis and (C) the multivariate analysis of oncogenic circRNAs in predicting the OS. Begg᾽s test for the pooled prognostic effect of tumor‐suppressive circRNAs in predicting the (D) OS and (E) DFS of oncogenic circRNAs in predicting the DFS of ESCC patients. (F) Visual Funnel plot of the pooled prognostic effect of oncogenic circRNAs in predicting the DFS of ESCC patients

Deek᾽s quantitative funnel plot was used to evaluate the publication bias among diagnostic studies, with a *p* value of = 0.215 (Figure 5A). Besides, Begg᾽s, Egger᾽s tests, and visual Funnel plot were adopted to appraise the bias among observation studies, and it was found that there was no inter‐study publication bias existing in the pooled diagnostic and prognostic effect sizes (Figure 5B–F), all with *p* > 0.05 for the Egger᾽s tests (data for Egger᾽s tests are not shown).

## 4 DISCUSSION

ESCC as one of the most common malignant tumors in the digestive tract is posing a threat to human health with a high mortality rate.^2^, ^3^, ^4^ Currently, surgical therapies combined with radiotherapy, chemotherapy, and other comprehensive treatments show somewhat improved resection rates and the 5‐year survival rate of EC. However, the 5‐year survival rate is still lower than 40%.^3^ On account of nontypical symptoms in early‐stage ESCC patients, they usually did not seek medical help until the advanced stage.^1^, ^2^ So they have missed the optimal time window for radical surgeries. CircRNAs are a group of newly found endogenous RNAs with coding/non‐coding functions and the absence of a 5᾽ end cap and a 3᾽ end poly A tail as well as the presence of a closed ring structure.^7^, ^9^, ^10^, ^11^, ^12^, ^14^, ^15^, ^16^ Such a special structure makes circRNAs highly conservative and stable.^7^, ^9^, ^10^, ^12^, ^13^, ^14^, ^15^ In recent years, it has been found that abnormalities in circRNA expression levels present high diagnostic and prognostic values in ESCC, which is, therefore, expected to be developed as biomarkers for the diagnosis and prognosis prediction of ESCC.^14^, ^15^, ^16^, ^17^, ^18^, ^19^, ^20^, ^21^, ^22^, ^23^, ^24^, ^25^, ^26^, ^27^ In this study, the application value of circRNA profiling in diagnosing and predicting the prognosis of ESCC has been systematically evaluated using the quantitative meta‐analysis.

Currently, a variety of meta‐analyses have reported the diagnostic efficacy of circRNAs in malignant tumors.^32^, ^33^, ^34^, ^35^ Wang, *et al*. have shown that the merged sensitivity, specificity and the AUC of circRNA in cancers are 0.72, 0.74, and 0.79, respectively.^35^ And our study has shown that the three indices in distinguishing ESCC from healthy controls using circRNA profiling are 0.78, 0.79, and 0.86 respectively. This indicates that circRNAs have high diagnostic values in ESCC. In addition, the merged PLR of 3.78 indicates that the possibility of abnormally expressed circRNAs in ESCC patients is about four times higher than that in matched controls. The merged NLR of 0.29 suggests that the false negative rate in the analysis of circRNA expressions is 29%. DOR is also an accurate index reflecting the diagnostic and detection efficiency, presenting an effective value between 1 and ∞. A DOR value of less than 1 indicates that the diagnostic and detection efficiency is very low.^36^ In this study, the merged DOR was 14.78. This indicates that the overall diagnostic efficiency is high. All this shows that circRNAs have promising values in the diagnosis of ESCC with high efficiency. Our findings are basically consistent with those in Niu᾽s study.^37^ In our study, the subgroup analysis has been carried out for investigating the association between expression levels of circRNAs and the sample size. It is found that the diagnostic efficiency of downregulated circRNAs is better than that of upregulated circRNAs. In addition, when the sample size is ≥70, the comprehensive efficiency of circRNA profiling in the diagnosis of ESCC can be significantly improved. However, due to the small sample size in the subgroup analysis, a possibility of bias exists. The conclusion needs to be confirmed in relevant studies with a large sample size.

At present, the efficacy of circRNA profiling in the prognosis evaluation of ESCC remains to be controversial. According to the cyclization mechanism of circRNAs, their exons may provide circRNA molecules with various biological functions. In this study, we have classified circRNAs in line with their biofunctions, and there is a negative correlation between oncogenic circRNA expressions and the prognosis of ESCC. The overexpressions of oncogenic circRNAs in ESCC patients are associated with the poor OS, while the overexpressions of tumor‐suppressive circRNAs improve the OS of ESCC patients. Specifically, patients with upregulations of oncogenic circRNAs present shorter OS than those with downregulated oncogenic circRNAs (HR = 3.24), while patients with upregulated tumor‐suppressive circRNAs present longer OS than those with downregulated ones (HR = 0.57). Li, et al. have reached a similar conclusion in the meta‐analysis of CRC,^31^ which further confirms the reliability of the results in our study.

The source of heterogeneity in this meta‐analysis is mainly caused by threshold effect and non‐threshold effect.^38^ The Spearman correlation coefficient analysis has shown that the heterogeneity in the overall merged statistics and the subgroup analysis mainly comes from the threshold effect that may result from different boundary values or cut‐off values. The difference in cut‐off value and internal reference genes used for relative quantification of circRNAs in the included studies can be one of the main reasons for heterogeneity. In the present study, we have further explored the possible factors that result in heterogeneity using the sensitivity and meta‐regression analyses. The sensitivity analysis shows that there are no deviant outliers, indicating that the homogeneity among the included studies is good. The meta‐regression analysis suggested that the sample size, circRNA signature, circRNA expression status, reference gene, cut‐off setting, and QUADAS scores were not likely to be the major factors that caused heterogeneity among the studies.

Besides, limitations in this study are as follows. First, the underlying population bias may exist in this study, and the merged effect size is based on the Asian population (mainly Chinese people). Second, the molecular type of included circRNAs and their sample types have not been unified, so the heterogeneity among the studies is large. Third, the sample size of included diagnostic studies is small, so the results are only for reference.

In conclusion, this study suggests that circRNA can be used as a promising auxiliary indicator for the diagnosis and prognosis monitoring of ESCC. However, our conclusion needs to be confirmed by more multi‐center, large‐sample‐size RCTs for late‐stage ESCC patients.

## CONFLICT OF INTEREST

None.

## Data Availability

The data that support the findings of this study are available from the corresponding author upon reasonable request.
